# E2F1: Cause and Consequence of DNA Replication Stress

**DOI:** 10.3389/fmolb.2020.599332

**Published:** 2021-02-16

**Authors:** Shahd Fouad, David Hauton, Vincenzo D'Angiolella

**Affiliations:** Department of Oncology, Medical Research Council Oxford Institute for Radiation Oncology, University of Oxford, Oxford, United Kingdom

**Keywords:** E2F1, DNA replication stress, ubiquitin proteasome system, cyclin F, retinoblastoma, cyclin E, ribonucleotide reductase

## Abstract

In mammalian cells, cell cycle entry occurs in response to the correct stimuli and is promoted by the transcriptional activity of E2F family members. E2F proteins regulate the transcription of S phase cyclins and genes required for DNA replication, DNA repair, and apoptosis. The activity of E2F1, the archetypal and most heavily studied E2F family member, is tightly controlled by the DNA damage checkpoints to modulate cell cycle progression and initiate programmed cell death, when required. Altered tumor suppressor and oncogenic signaling pathways often result in direct or indirect interference with E2F1 regulation to ensure higher rates of cell proliferation independently of external cues. Despite a clear link between dysregulated E2F1 activity and cancer progression, literature on the contribution of E2F1 to DNA replication stress phenotypes is somewhat scarce. This review discusses how dysfunctional tumor suppressor and oncogenic signaling pathways promote the disruption of E2F1 transcription and hence of its transcriptional targets, and how such events have the potential to drive DNA replication stress. In addition to the involvement of E2F1 upstream of DNA replication stress, this manuscript also considers the role of E2F1 as a downstream effector of the response to this type of cellular stress. Lastly, the review introduces some reflections on how E2F1 activity is integrated with checkpoint control through post-translational regulation, and proposes an exploitable tumor weakness based on this axis.

## Introduction

Central to the homeostasis of a functioning organism is the capacity of cells to divide. This is achieved in progressive phases collectively known as the cell cycle. Individual cells replicate their DNA (S phase) and then separate it into daughter cells (M phase). Gap phases (G_1_ and G_2_) temporally segregate these two events. The correct entry into and execution of each phase is ensured by the presence of cell cycle checkpoints which guarantee error-free completion of the process of cell division (Hartwell and Kastan, 1994). Cyclins, with their cyclin-dependent kinase (Cdk) partner proteins, are periodically expressed during the cell cycle to promote cell cycle progression. Cyclin Ds assemble with Cdk4/6 to promote G_1_/S transition, progression through S phase is stimulated by cyclin E associating with Cdk2, and transition from G_2_ to M phase is driven by cyclin A/B partnered with Cdk1 (Deshpande et al., 2005). Unlike other cyclins, cyclin F does not bind to or activate any Cdks (Fung et al., 2002; D'Angiolella et al., 2010). Instead, cyclin F has an F-box domain which is essential for its activity as an E3 ubiquitin ligase. This F-box domain mediates the interaction of cyclin F with Skp1, which in turn binds to Cullin1 (Cul1), in order to form the Skp1–Cul1–F-box multi-subunit ubiquitin ligase complex SCF^Cyclin F^. Through its E3 complex, cyclin F promotes the ubiquitylation of multiple substrates, many of which are directly involved in DNA replication (D'Angiolella et al., 2010, 2012; Emanuele et al., 2011; Elia et al., 2015; Walter et al., 2016; Burdova et al., 2019; Clijsters et al., 2019).

DNA damage checkpoints control the cell cycle by inhibiting Cdk activity thereby preventing cell cycle phase transitions when necessary. This is required in the case of incomplete DNA replication or in the presence of unrepaired DNA damage, to which the checkpoint responds by maintaining a low activity level of S and M phase Cdks. Events that obstruct the process of DNA replication elicit a specialized process, known as the DNA damage checkpoint response, which involves amplifying the DNA damage signals from ataxia telangiectasia and rad3-related (ATR) and ataxia-telangiectasia mutated (ATM) kinases (the *sensors*) by the checkpoint 1 and 2 (Chk1 and Chk2) kinases (the *transducers*) (Bartek and Lukas, 2003). By the conventional distinction, ATR recognizes single-stranded DNA (ssDNA) whilst ATM recognizes double-strand breaks (DSBs) (Branzei and Foiani, 2010). Additional stimuli beyond ssDNA and DSBs can also activate ATR and ATM, and activation of these sensor kinases is an intense area of investigation. Following phosphorylation by ATR and ATM, Chk1 and Chk2 initiate signaling cascades that include an activating phosphorylation on the kinase Wee1 (Lee et al., 2001), and an inhibitory phosphorylation on the phosphatase Cdc25 (Busino et al., 2003; Watanabe et al., 2004). The inhibition of Cdc25 further facilitates the activity of Wee1 which phosphorylates Cdks on Tyr15, thereby inhibiting Cdk activity and arresting cell cycle progression (Watanabe et al., 2004).

In addition to the DNA damage checkpoint control, the DNA replication process is divided into discrete steps whose tight regulation ensures DNA replication fidelity. First, pre-replication complexes (pre-RCs) assemble at DNA replication origins in the G_1_ phase of the cell cycle, this process is known as origin licensing. This is followed by replication initiation at the G_1_/S phase transition, where Cdks and Ddks (DBF4-dependent kinases) activate the mini-chromosome maintenance (MCM) helicases, allowing DNA unwinding and recruitment of Cdc45 and GINS to form the active CMG (Cdc45-MCM-GINS) complex. Bidirectional DNA synthesis initiation follows (Diffley, 2011). Finally, DNA replication is terminated through the convergence of two replication forks followed by removal of the MCM helicases from the DNA (D'Angiolella and Guardavaccaro, 2017; Dewar et al., 2017; Sonneville et al., 2017). The DNA replication programme is susceptible to impediments to DNA synthesis and such events are widely ascribed as causes of a broad range of cellular stresses known collectively as DNA replication stress. A clear-cut standard definition for DNA replication stress is difficult to formulate because it is an umbrella term that encompasses many replication defects, and the list of such defects is continuously evolving. One commonly accepted way to describe DNA replication stress is to define it as the transient hindering of DNA replication which often includes stalled/collapsed replication forks and/or dysregulated replication origin firing. Accumulated ssDNA and DNA DSBs are considered markers of this type of stress. DNA replication stress is implicated in the process of cancer development and has even been proposed as an initiation event for tumorigenesis (Halazonetis et al., 2008) from which two hallmarks of cancer derive, namely genomic instability and evading programmed cell death (Macheret and Halazonetis, 2015).

Cell cycle progression, leading to DNA replication and cell division, is orchestrated by a wave of transcriptional activation which, coupled to ubiquitylation, guarantees irreversible cell cycle phase transitions. The E2F family of transcription factors has major roles in cell cycle control, DNA replication and repair, apoptosis, checkpoint response, development and differentiation (reviewed in Meng and Ghosh, 2014). This protein family comprises the transcriptional activators E2F1/2/3A, the canonical transcriptional repressors E2F3B/4/5/6 and the atypical transcriptional repressors E2F7/8 (Chen et al., 2009). The levels of activators and atypical repressors fluctuate throughout the cell cycle, peaking at the G_1_/S transition and late S phase respectively, while the canonical repressors remain stably expressed during all cell cycle phases (Chen et al., 2009; Kent and Leone, 2019). E2F transcriptional activators promote the transcription of numerous genes required for S phase entry and DNA synthesis. In fact, E2F-dependent transcription was recently found to determine the DNA replication capacity, which is the amount of DNA a cell can synthesize per unit time (Pennycook et al., 2020). Ubiquitylation events mediated by SCFs and the anaphase promoting complex/cyclosome (APC/C) promote the degradation of E2Fs before mitosis, thereby resetting the cell cycle (reviewed in Emanuele et al., 2020).

This review summarizes the role of the archetypal member of the E2F family – namely E2F1 – in the DNA replication stress pathway and highlights its dysregulation in cancer. The review preferentially focuses on E2F1 as it is the most thoroughly studied member of the E2F family. Further, hyper-proliferation effects in cancer have been directly ascribed to E2F1 dysregulation (Yee et al., 1987; Attwooll et al., 2004), and E2F pro-apoptotic activity is mainly attributed to E2F1 (DeGregori et al., 1997; Kent and Leone, 2019). This manuscript reviews the multiple oncogenic pathways that converge on aberrantly altering E2F1 functions. These alterations ensure continuous cell cycle progression independently of external stimuli. In addition, given the crucial transcriptional contribution of E2F1 to DNA replication, the constitutive activity of E2F1 is a plausible reason for the build-up of DNA replication stress. Finally, a strong regulatory link exists between Chk1 and E2F1 which underlines the necessity of maintaining checkpoint control while E2F1 activity is high. Regulation of E2F1 in checkpoint-proficient and checkpoint-defective cells is discussed here and an associated potential genetic dependency with clinical implications is highlighted.

## Oncogenic Signaling Pathways Converging On E2F1 Dysregulation

One of the well-established hallmarks of cancer is dysregulated cell cycle progression (Hanahan and Weinberg, 2011). Cancer cells often acquire mutations that disrupt the control of the transcriptional programme responsible for cell cycle entry. These mutations allow cancer cells to become refractory to the external stimuli that are normally required for G1 entry, which in turn enables cells to replicate indefinitely. Commonly mutated oncogenes and tumor suppressors that converge on aberrant E2F activation are summarized below.

### Retinoblastoma (Rb)

**Figure 1:**
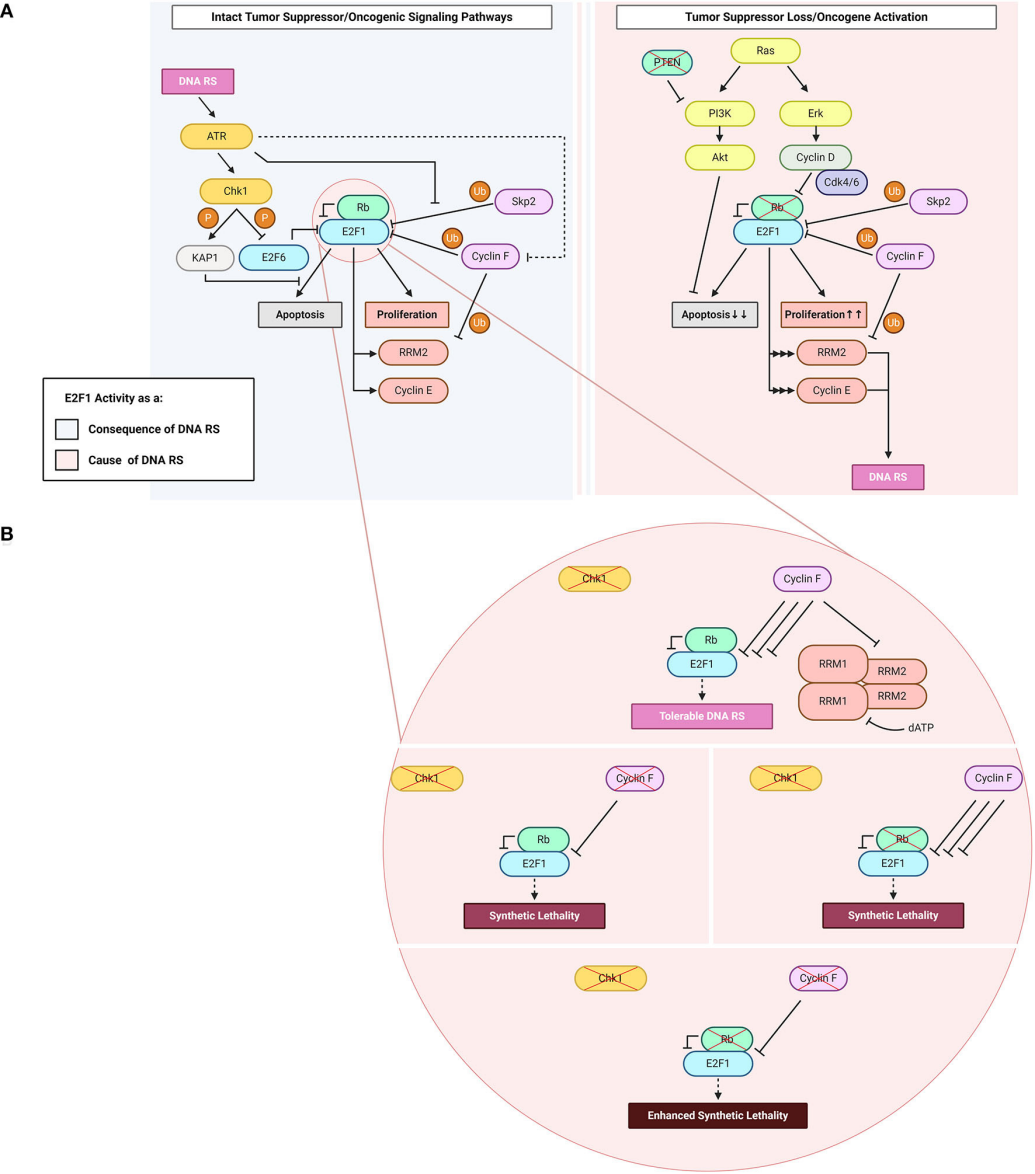
E2F1: roles as a cause and a consequence of DNA replication stress (“DNA RS” in figure). **(A)** E2F1 functions upstream and downstream of DNA replication stress. Highlighted in the figure are E2F1-engaging pathways downstream of the DNA RS response when oncogenic and tumor suppressor signaling pathways are intact (*light blue*), and upstream of DNA RS when oncogenic and/or tumor suppressor signaling pathways are altered (*light pink*). **(B)** Consequences of abrogating the E2F1 post-translational regulatory triad comprising Chk1, Rb, and cyclin F. *Created with BioRender.com*.

The product of the gene *RB1*, retinoblastoma (Rb), is a potent tumor suppressor. Loss of *RB1* in the germline underlies the development of eye cancer in children. The main function of Rb is to modulate the transcriptional activity of E2F1 (Figure 1) (Frolov and Dyson, 2004). E2F1, like other typical transcription factors in the E2F family, has a DNA-binding domain (DBD) in addition to a dimerization domain through which it associates with dimerization partner (DP) proteins. E2F transcriptional activators partnered with DP proteins activate gene expression (DeGregori and Johnson, 2006), and the association of Rb with E2F1 blocks this trans-activating domain of the E2F1-DP complex. Phosphorylation of Rb by Cdks in early as well as late G_1_ phase leads to the release of E2F1 from this transcriptionally-repressive complex, and hence allows it to promote the transcription of genes whose protein products are required for cell cycle entry and G_1_/S cell cycle transition (La Thangue, 1994; Wu et al., 2001; Polager et al., 2002).

Since cell cycle and DNA synthesis proteins are among the main transcriptional targets of E2F1, mutations in the Rb/E2F1 axis allow aberrant cell proliferation preferred by cancer cells (Figure 1). Such mutations are frequent in cancer to the extent that the dysregulation of this axis and the resulting continuous activity of E2F1 are described in almost all human malignancies (Attwooll et al., 2004). Very early evidence suggested that perturbations in this pathway may result in DNA replication stress (Bartkova et al., 2005), although very few studies have specifically addressed DNA replication stress ensuing from E2F1 dysregulated activity.

### Ras

The mitogen-activated protein kinase (MAPK) pathway is an important signaling pathway that lies downstream of receptor tyrosine kinases (RTKs) and transduces extracellular growth-promoting signals that are received by RTKs such as epidermal growth factor receptors (EGFRs) (Downward, 2003). Following RTK activation, son of sevenless (Sos) is recruited to the plasma membrane *via* interacting with Grb2. Sos is a guanine nucleotide exchange factor (GEF) which promotes the activation of membrane-bound Ras by catalyzing the exchange of Ras-bound guanosine diphosphate (GDP) for guanosine triphosphate (GTP) (Downward, 2003). Active (GTP-bound) Ras can then amplify the signaling cascade through downstream MAPK pathway components such as extracellular signal-regulated kinase (ERK). ERK can directly phosphorylate cyclin D and promote its activation (Lavoie et al., 1996; von Willebrand et al., 2003). Hyper-active cyclin Ds, in complex with Cdk4/6, phosphorylate Rb which, in turn, leads to activation of E2F1 transcriptional activity. The loss of negative E2F1 regulation permits continuous entry into the cell cycle (Figure 1A). *RAS* is among the most frequently mutated genes in human malignancies (Ahearn et al., 2011). The majority of mutations reside on the G12 residue and these mutations promote the constitutive binding of Ras to GTP, and thus the continuous activation of Ras (Hobbs et al., 2016).

Ras-activating mutations could result in E2F1 hyper-activity and trigger DNA replication stress which furthers cancer transformation. It is important to mention that the ERK-E2F1 relationship is not unidirectional; E2F1 also regulates ERK through trans-activating Ras guanyl releasing protein 1 (RASGRP1) and RasGEF domain family member 1B (RASGEF1B), two GEFs that promote the activation of Ras and consequently its downstream effector ERK (Mitin et al., 2005; Korotayev et al., 2008). It was also found that the regulation of ERK by E2F1 is necessary for E2F1-induced G_1_/S cell cycle transition, underscoring the importance of this feedback (Korotayev et al., 2008). Another positive feedback loop reported between E2F1 and Ras is mediated by integrin-linked kinase (ILK). Ras promotes ILK expression through E2F1, which acts as an effector in this loop. In return, ILK relaxes the G-quadruplex structure in the promoter region of Ras, which promotes higher Ras transcription (Kalra et al., 2010; Chu et al., 2016). Thus, in cancer the dysregulation of E2F1 and/or Ras results in an auto-amplification loop which promotes proliferation and facilitates cancer transformation. Such hyper-activation of E2F1 could also underlie the significant accumulation of DNA replication stress that represents a barrier to cancer progression if it occurs in the early stages of transformation. This barrier to oncogenic transformation has evolved so that oncogene expression in human cells results in senescence, whereby cells are unable to replicate but survive in a “senescent” state. Senescence relies on the control of E2F1 activity by the Cdk inhibitors p15 and p16, which block the function of Cdk4/6 and thereby suppress E2F1 activity (Serrano et al., 1997; Malumbres et al., 2000). Indeed, loss of p16 is a common mutation in cancer and it enables cells to evade Ras-induced senescence and promotes malignant transformation (Tanaka et al., 1994; Serrano et al., 1996).

### Phosphoinositide 3-Kinase (PI3K)

RTKs lie upstream of several pathways including the phosphoinositide 3-kinase (PI3K)/Akt pathway. In response to their ligand-dependent phosphorylation, EGFRs are bound by class 1a PI3Ks *via* Src homology 2 (SH2) domains. This leads to the activation of PI3K and the subsequent phosphorylation of phosphatidylinositol 4,5-bisphosphate (PIP_2_) to generate phosphatidylinositol 3,4,5-triphosphate (PIP_3_) (Cantley, 2002; Hoxhaj and Manning, 2020). *Via* its pleckstrin homology (PH) domain, Akt binds PIP_3_ and is then phosphorylated and thus activated by phosphoinositide-dependent protein kinase-1 (PDK-1). This is followed by additional Akt phosphorylation through mechanistic target of rapamycin complex 2 (mTORC2) to further promote Akt activity (Cantley, 2002; Hoxhaj and Manning, 2020). The PI3K/Akt pathway is a central pathway often dysregulated in cancer through constitutive activation.

E2F1-mediated transcription promotes apoptosis in addition to cell cycle progression (Figure 1A). Numerous circuits have been proposed to control the balance of pro-survival vs. pro-apoptotic activity of this transcriptional activator, and there are excellent reviews on the topic (see Polager and Ginsberg, 2009; Engelmann and Pützer, 2012; Kent and Leone, 2019). The PI3K/Akt pathway potently inhibits E2F1-mediated apoptosis by repressing multiple pro-apoptotic genes that are transcriptional targets of E2F1 (Hallstrom and Nevins, 2003; Hallstrom et al., 2008; Pützer and Engelmann, 2013). To this end, Akt is involved in a negative feedback loop with E2F1 through Grb2-associated binder 2 (Gab2), which is downstream of the transcriptional activity of E2F1 (Chaussepied and Ginsberg, 2004). Grb2 relays mitogenic signals from cell-surface receptors to PI3K and Akt and, in turn, this E2F1-promoted Akt activation inhibits E2F1-mediated apoptosis (Hallstrom and Nevins, 2003; Chaussepied and Ginsberg, 2004). One mechanism by which Akt suppresses the pro-apoptotic activity of E2F1 is through topoisomerase IIβ-binding protein 1 (TopBP1). Akt mediates TopBP1 phosphorylation, and the phosphorylated protein suppresses the transcription of E2F1 pro-apoptotic target genes (Liu et al., 2006). The Rb-independent suppression of E2F1 pro-apoptotic activity takes place during normal G_1_/S transition and in response to DNA damage, and involves the recruitment of Brg1/Brm – an integral unit of the SWItch/sucrose non-fermentable (SWI/SNF) chromatin-remodeling complex – to E2F1 target promoters (Liu et al., 2004, 2006). Since this function of TopBP1 is specific to E2F1 but not to other E2F transcriptional activators, and E2F pro-apoptotic activity is mainly credited to E2F1 (DeGregori et al., 1997; Kent and Leone, 2019), this mechanism essentially allows S phase entry whilst also inhibiting apoptosis (Liu et al., 2004, 2006). Overall, the constitutive activation of the PI3K/Akt pathway in tumors favors the pro-survival and DNA replication promoting activities of E2F1 (Figure 1A).

## E2F1 Dysregulation as a Cause of DNA Replication Stress

Amongst E2F1 transcriptional targets are genes that are essential for the process of DNA replication. Whilst physiological levels of active E2F1 allow the execution of DNA replication, it has been shown that increased E2F1 activity could predispose cells to the acquisition of DNA replication stress (Bartkova et al., 2005; Liontos et al., 2009; Bester et al., 2011; Burdova et al., 2019). Mechanisms promoting DNA replication stress stemming from E2F1 dysregulation, however, are less studied. Highlighted below are the potential mechanisms underlying DNA replication stress induced by E2F1 through its known transcriptional targets.

### E2F1 Transcriptional Target: Cyclin E

**Figure 2:**
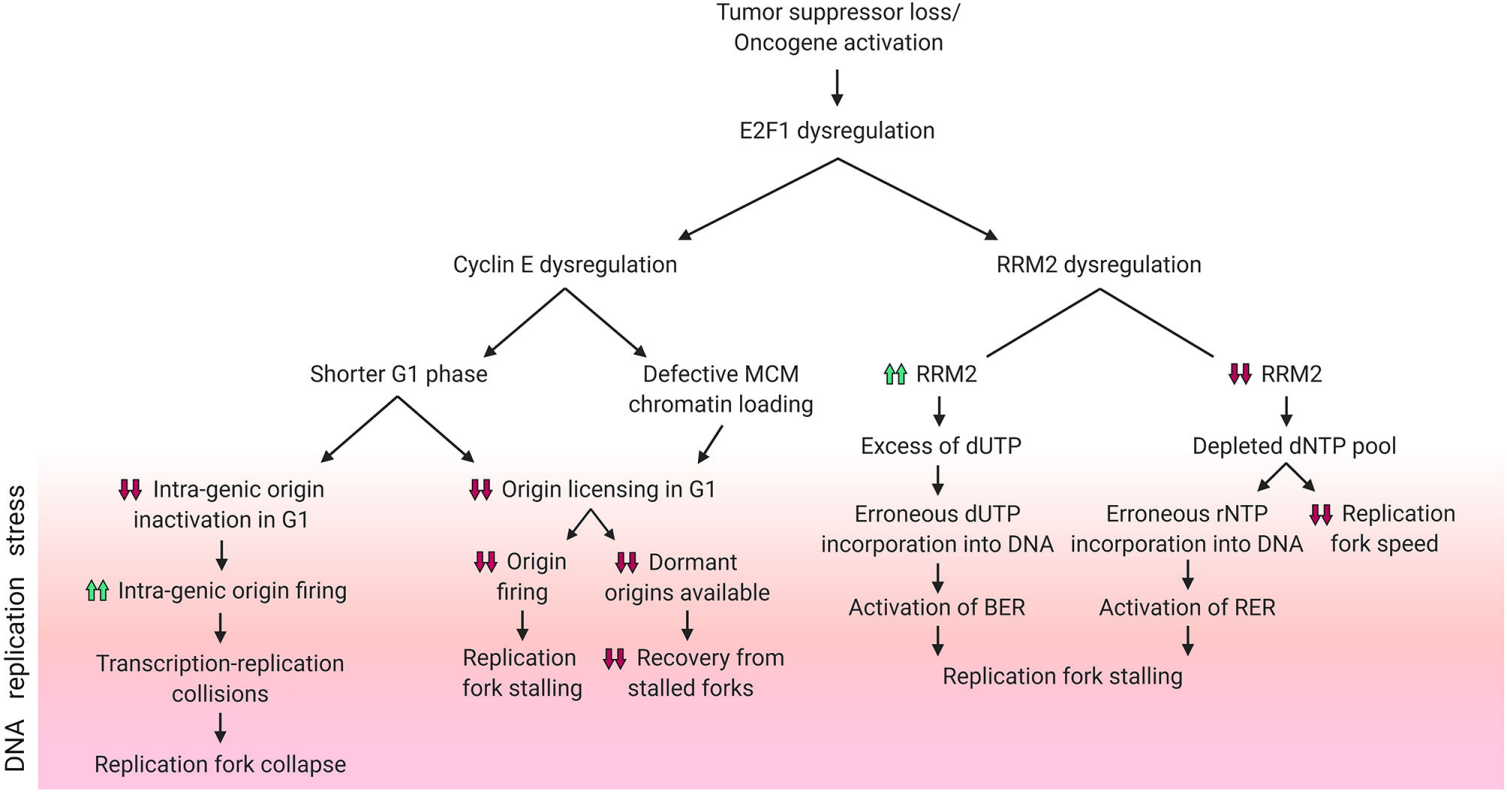
DNA replication stress phenotypes resulting from E2F1 dysregulation. Schematic diagram summarizing the different DNA replication stress phenotypes caused by dysregulation of E2F1 and its transcriptional targets cyclin E and RRM2. Phenotypes included above for cyclin E are reported in the literature, while those for RRM2 are hypothesized. *Created with BioRender.com*.

Cyclin E is a transcriptional target of E2F1 that contributes to DNA replication stress. In complex with Cdk2, cyclin E promotes the phosphorylation of essential DNA replication factors in order to initiate and allow the progression of bidirectional DNA synthesis (Zhang, 2007). Cyclin E can be regulated independently of E2F1 as well: cyclin E overexpression, a common event in cancer, can arise from its defective proteolysis through inactivating mutations in its E3 ubiquitin ligase Fbxw7 (Caruso et al., 2018). Additionally, direct amplification of the *CCNE* gene locus is observed in ovarian (22%), esophageal/gastric (18%), bladder (7%), and pancreatic (6%) cancers (Caruso et al., 2018). Further, given that E2F1 lies upstream of cyclin E (Inoshita et al., 1999), it is easy to see how E2F1 overexpression can be reflected in cyclin E hyper-activity. Owing to the widespread dysregulation of cyclin E in cancer, numerous studies have established a direct role for this cyclin in DNA replication stress. The mechanisms underlying cyclin E overexpression-induced stress are debated and there is contrasting evidence depending on the models being used. The main source of replication stress in these studies is ascribed to the defective modulation of DNA replication origins, however some groups report under-firing (Resnitzky et al., 1994; Ekholm-Reed et al., 2004) whilst others report over-firing (Jones et al., 2013; Macheret and Halazonetis, 2018) of replication origins in response to aberrantly high cyclin E levels (Figure 2).

Lower rates of origin firing can result from reduced replication origin licensing, a process which occurs in G_1_ (Figure 2). Following the overexpression of the G_1_/S transition-promoting protein cyclin E, the G_1_ phase is shortened, which in turn hinders replication origin licensing (Resnitzky et al., 1994; Ekholm-Reed et al., 2004). Reduced origin licensing could have multiple adverse effects on the DNA replication programme. One example is the need for the reduced number of replication forks to travel further in order to cover the same genomic area that is covered by the larger number of forks in unstressed cells (Hills and Diffley, 2014). This increased pressure on the limited number of replication forks magnifies the risk of under-replicated DNA and of the stalling of these forks (Hills and Diffley, 2014). Another harmful effect of a decreased rate of origin licensing is the subsequent decrease in the availability of back-up origins, known as dormant origins. Dormant origins have the potential for rescuing stalled replication forks through acting as back-up origins from which new forks could start (Blow et al., 2011). Additionally, defective chromatin-loading of the origin licensing helicase complex MCM2-7 can also contribute to a cyclin E-dependent reduction in origin firing (Ekholm-Reed et al., 2004).

As mentioned above, other studies have reported increased origin firing upon cyclin E overexpression (Figure 2). Using models of induced overexpression of cyclin E in U2OS cells it was shown that, in contrast to normal cells which space their DNA replication origins in between genes (inter-genic) to avoid conflicts between the transcription and replication machineries, new origins in cyclin E overexpressing cells were found within genes (intra-genic). This reportedly occurs due to the lack of time in G_1_ for the transcription machinery to inactivate intra-genic origins (Macheret and Halazonetis, 2018). This higher rate of origin firing results in transcription-replication collisions and an ensuing collapse of forks originating from cyclin E-induced intra-genic origins (Macheret and Halazonetis, 2018). Replication stress driven by transcription-replication collisions can be alleviated through the inhibition of either transcription elongation or origin firing (Jones et al., 2013; Macheret and Halazonetis, 2018). In addition, cyclin E-induced replication stress in the form of fork slowing can also result from the replication machinery colliding with R-loops (Jones et al., 2013). R-loops are 3-stranded RNA:DNA hybrid structures that include a displaced DNA single strand, and such structures can be resolved using RNase H1 enzyme which specifically degrades RNA in these complexes. Through transfecting RNase H1 into cyclin E overexpressing cells, Jones et al. showed a partial rescue of fork speed (Jones et al., 2013). Literature shows that R-loop formation can be directly promoted by Ras activation (Kotsantis et al., 2016). It can be speculated that the sequence of events leading to this reported phenotype is: Ras hyper-activates E2F1, which increases the transcription of cyclin E, which in turn promotes the formation of R-loops and finally results in DNA replication stress.

Similarly to studies of cyclin E hyper-activity, models of hyper-activity of its upstream transcriptional activator E2F1 also show resultant DNA damage. An important example is a model of E2F hyper-activation promoted by the loss of the E2F repressor E2F6 (Pennycook et al., 2020). In this model, the authors used siRNA against E2F6 to study the consequences of E2F activation in S phase. The authors report an increased speed of DNA replication but an unaltered number of active origins. The increase in DNA replication speed resulted in the formation of DSBs which triggered a DNA damage response in the second cell cycle after E2F6 siRNA, suggesting a different mechanism than the one reported for DNA replication stress ensuing from cyclin E overexpression (Pennycook et al., 2020). Interestingly, loss of E2F1 and E2F2 in mice resulted in DNA damage (Iglesias-Ara et al., 2015). The mechanism underlying increased DNA damage upon E2F1 and E2F2 loss is unclear but this, together with the aforementioned findings, makes a stronger case for the importance of E2F1 homeostasis and the restriction of E2F1 hyper-activity.

### E2F1 Transcriptional Target: RRM2

The evidence mentioned above suggests there are several mechanisms of DNA replication stress promoted by E2F1 dysregulation. An important transcriptional target of E2F1 which could contribute to DNA replication stress is ribonucleotide reductase member 2 (RRM2) (DeGregori et al., 1995; Chabes et al., 2004). RRM2 is the regulatory subunit of the essential enzyme ribonucleotide reductase (RNR). RNR is required for the reduction of ribonucleotide diphosphates (rNDPs) to deoxyribonucleotide diphosphates (dNDPs), a rate-limiting step in the catalysis of the synthesis of deoxyribonucleotide triphosphates (dNTPs). RNR is a hetero-tetramer, composed of two homodimers formed by RRM1 (large/catalytic subunit) and RRM2 (small/regulatory subunit). RRM2 is cell cycle regulated and its synthesis is transcriptionally promoted by E2F1 at the G_1_/S phase transition (DeGregori et al., 1995; Chabes et al., 2004). At the end of S phase, RRM2 is selected for ubiquitin-mediated proteolysis by the E3 ubiquitin ligase cyclin F (Figure 1A) (D'Angiolella et al., 2012).

These mechanisms ensure that dNTP production is strictly coupled to DNA replication progression. Imbalances between DNA replication progression and dNTP production are potential causes of DNA replication stress. Indeed, it was shown that aberrantly increased activation of E2F1 through the overexpression of the viral oncogenes HPV E6/E7 (which inactivate Rb) promotes hyper-proliferation, but fails to parallel this with sufficient activation of dNTP synthesis genes. Thus, expression of E6/E7 and the consequent activation of E2F1 results in a reduction of the dNTP pool (Reichard, 1988; Maya-Mendoza et al., 2015) and hence DNA replication stress (Bester et al., 2011). Hyper-active E2F1 increased the gene expression of *RRM2*, but it failed to increase the expression of 4 other genes that are important for dNTP synthesis – namely *IMPDH2, NME1, DHODH, PPAT* (Bester et al., 2011) – leading to dNTP depletion.

To ensure a balanced and sufficient pool of dNTPs is available during DNA replication, RNR is subject to negative allosteric regulation by one of its dNTP products: dATP. The activity regulatory site (a-site) of RNR binds either activator ATP or inhibitor dATP molecules, and these allosteric regulators switch RNR on or off, respectively (Figure 1B) (Brown and Reichard, 1969; Nordlund and Reichard, 2006). This ensures the presence of adequate yet not excessive levels of dNTPs. In addition to the a-site, RNR also has a specificity site (s-site) which switches RNR activity preferentially toward different nucleotides (Brown and Reichard, 1969; Nordlund and Reichard, 2006). It is important to note that RNR reduces ADP, CDP, GDP, and UDP – but not TDP – and the resultant dNDPs are then phosphorylated by the nucleotide diphosphate kinase to dNTPs. Converting dUTP to dTDP is carried out by other enzymes, namely dUTPase (dUDP to dUMP), thymidylate synthase (dUMP to dTMP) and thymidylate kinase (dTMP to dTDP) (Reichard, 1988). Due to the lack of direct dTDP generation by RNR, it is possible to speculate that elevated RNR levels following RRM2 dysregulation by E2F1 might contribute to DNA replication stress and genomic instability by creating an excess of dUDP – which upon further phosphorylation could be erroneously incorporated into the DNA thereby triggering the activation of the base excision repair pathway (BER) – if the dTDP synthesis enzymes were not upregulated in parallel (Figure 2). In fact, it has been shown that high expression of RRM2 impedes thymidine kinase-mediated DNA repair by contributing to dUTP incorporation (Hu et al., 2012a). However, since E2F1 also regulates dUTPase which hydrolyzes dUTP/dUDP to dUMP (DeGregori et al., 1995; Banerjee et al., 1998; Wilson et al., 2009), it is possible that the effect of excess dUDP is canceled out by dUTPase upregulation. An alternative method by which RRM2 dysregulation by E2F1 might contribute to DNA replication stress is through dNTP pool depletion resulting from attenuated trans-activation of RRM2. The insufficient levels of dNTPs could result in decreased replication fork speed and would possibly favor the incorporation of ribonucleotides into the DNA, with a potential concurrent extensive activation of ribonucleotide excision repair pathway (RER) (Figure 2).

Low levels of dNTPs have been linked to DNA replication stress in early precancerous lesions (Bester et al., 2011), while aberrantly high RRM2 expression correlates with poor cancer prognosis (Ferrandina et al., 2010; Morikawa et al., 2010a,b; Satow et al., 2010; Jones et al., 2011; Kretschmer et al., 2011). Thus, studies investigating alterations in the E2F1-RRM2 axis are necessary to establish the exact mechanisms underlying DNA replication stress in early and late stages of cancer progression.

## E2F1 Regulation as a Consequence of DNA Replication Stress

Aberrant accumulation or hyper-activity of E2F1 can contribute to DNA replication stress phenotypes (Bartkova et al., 2005; Liontos et al., 2009; Bester et al., 2011; Burdova et al., 2019). At the same time, E2F1 activity can prevent the adverse development of DNA replication stress into DNA damage (Bertoli et al., 2013a, 2016). It can be speculated that these opposing functions are due to the presence of an intact checkpoint response in conditions of E2F1 hyper-activity. Summarized below are the regulation and functions of E2F1 in cells with either proficient or defective checkpoint responses.

### E2F1 Regulation in Checkpoint-Proficient Cells

Upon encountering stress imposed onto the DNA replication machinery by internal or external factors, the cell activates the DNA damage checkpoint response in order to both repair the DNA damage and activate programmed cell death in situations where the DNA damage is irreparable. E2F1 plays crucial roles in the checkpoint response; it contributes to both restarting the cell cycle and initiating apoptosis. The checkpoint kinase Chk1 can activate E2F1 in order to promote the transcription of RRM2 (Zhang et al., 2009). This process ensures that RNR produces enough dNTPs for DNA repair to be completed. There is further evidence highlighting the necessity of sustaining the activity of transcriptional activators of the E2F family, including E2F1, for the implementation of a proper DNA damage checkpoint response (Bertoli et al., 2013a, 2016). This sustained activation is carried out by a conserved mechanism of replication stress-induced deactivation of the negative feedback loop imposed by the transcriptional repressor E2F6, an E2F target itself, on its fellow E2F family members (Bertoli et al., 2013a,b). Chk1 is responsible for the deactivating phosphorylation on E2F6 in response to DNA replication stress, thereby promoting E2F transcriptional activity and hence E2F trans-activation of DNA repair genes (Figure 1A) (Bertoli et al., 2013a).

In response to extensive DNA damage, E2F1 also trans-activates pro-apoptotic genes through the ATR-Chk1-E2F1 axis (Liu et al., 2000). In these conditions of cellular stress, E2F1 could switch from promoter of cell cycle progression to promoter of apoptosis. In cases where the extent of DNA damage is not extensive, the checkpoint response attempts to repair the damage instead of initiating programmed cell death. To this end, the checkpoint response hinders the pro-apoptotic functions of E2F1 the Krüppel-associated box-associated corepressor 1 (KAP1). KAP1 is phosphorylated by ATR/Chk1 on S473 in order to promote its binding to the marked-box domain of E2F1, a domain through which E2F1 executes its pro-apoptotic functions (Figure 1A) (Hallstrom and Nevins, 2003; Wang et al., 2007; Hu et al., 2012b). In addition to interacting with the marked-box domain of E2F1, which is critical for the accumulation of both p53 and p73, KAP1 mediates the recruitment of histone deacetylase 1 (HDAC1). HDAC1 impedes the acetylation of E2F1, a post-translational modification that is known to promote its DNA-binding capacity (Martínez-Balbás et al., 2000) and is required for E2F1 recruitment onto promoters of pro-apoptotic E2F1 target genes in response to genotoxic stress (Pediconi et al., 2003).

The ubiquitin-proteasome system (UPS) is a crucial player in the process of E2F1 regulation. ATR and ATM – the DNA replication stress and DNA damage sensor kinases, respectively (Branzei and Foiani, 2010) – can induce E2F1 phosphorylation on S31 (Lin et al., 2001). S31 phosphorylation increases E2F1 half-life through generating a binding motif for 14-3-3τ on E2F1, thereby inhibiting E2F1 ubiquitylation (Wang et al., 2004). E2F1 is degraded by the E3 ligase SCF^Skp2^, a process during which Skp2 binds to the first 41 amino acids of E2F1 among which S31 lies (Figure 1A) (Marti et al., 1999; Wang et al., 2004). This stabilization of E2F1 is followed by its acetylation in instances where the DNA insults are significant, for example in response to treatment with high doses (μM range) of the DNA-intercalating and topoisomerase II-inhibiting drug doxorubicin. The hindering of UPS-mediated proteolysis of E2F1 ensures that E2F1 triggers apoptosis in cases where the DNA damage is irreparable (Pediconi et al., 2003). However, this pro-apoptotic response is not recapitulated when the cells encounter less deleterious forms of DNA replication interference such as UV-B irradiation-induced fork stalling (Pediconi et al., 2003), and is antagonized through S473-phosphorylated KAP1 following treatment with lower doses (nM range) of doxorubicin (Hu et al., 2012b). The regulation of E2F1 by the checkpoint after DNA damage is complex and needs to be comprehensively analyzed to understand whether diverse mechanisms of regulation are based on the type and intensity of DNA damage and lead to different outcomes.

### E2F1 Regulation in Checkpoint-Defective Cells

In line with the evidence presented above on how the checkpoint-mediated phosphorylation of E2F1 protects it against proteolysis, a novel mechanism of E2F1 regulation after checkpoint inhibition was recently identified. The study, conducted in our laboratory, shows that E2F1 is degraded upon treatment with Chk1 inhibitors, and that the E3 ligase cyclin F is the main mediator of this proteasomal degradation (Burdova et al., 2019). In these settings of Chk1 inactivity, the concomitant depletion of cyclin F results in E2F1 accumulation and a consequent DNA replication catastrophe that is followed by cell death (Figure 1B). The exact mechanism of how E2F1 triggers cell death in these settings is not clearly understood (Burdova et al., 2019). While cyclin E dysregulation was excluded as a possible mechanism in these models, preliminary data from our laboratory suggests that dNTP depletion and transcription-replication collisions could play a role. Since the loss of cyclin F also leads to the stabilization of RRM2, the depletion of dNTPs is counterintuitive in models where cyclin F is knocked-out. Likely, the low levels of dNTPs are due to increased consumption by the replication machinery due to E2F1 hyper-activity. Importantly, it has been shown that in models of Rb loss with high E2F1 activity, inhibition of Chk1 results in cell death through a mitotic catastrophe (Figure 1B) (Witkiewicz et al., 2018). This observation mirrors the cyclin F loss-Chk1 inhibition *synthetic lethality*, and the mitotic phenotypes require further investigation after cyclin F loss (Hartman et al., 2001).

The aforementioned evidence allows one to speculate that Rb, cyclin F and Chk1 may constitute a triad that regulates E2F1 post-translationally; when one of the triad components is defective (e.g., Chk1), another's (e.g., cyclin F's) E2F1-regulatory activity increases to compensate for it, thereby the ensuing DNA replication stress is limited to levels tolerated by the cell (Witkiewicz et al., 2018; Burdova et al., 2019). However, when two (e.g., cyclin F and Chk1) out of three components are defective, the consequences for the cell are not compensable and the net effect is a DNA replication catastrophe (Witkiewicz et al., 2018; Burdova et al., 2019). Hence, one could envision the phenotype of *enhanced* synthetic lethality to result from all three triad components being defective (Figure 1B). Enhanced synthetic lethality is an appealing approach used to further the clinical benefit of synthetic lethality by using additional approaches that target converging molecular mechanisms (Boshuizen and Peeper, 2020). The hypothesis introduced here of parallel pathways potentially controlling E2F1 upon checkpoint inhibition highlights the therapeutic potential of using Chk1 inhibitors in tumors with defective Rb or cyclin F, or – better yet – both. The co-dependence in E2F1 regulation between cyclin F & Chk1 and that between Rb & Chk1 have been reported in the literature (Witkiewicz et al., 2018; Burdova et al., 2019), and although the speculated Rb & cyclin F link has not been experimentally proven, according to cBioPortal's combined study (from ~45,000 samples) the loss of both Rb and cyclin F together occurs with a frequency that is ~30-fold lower than the loss of either gene alone (Cerami et al., 2012; Gao et al., 2013). Further studies of this axis could help foster a better understanding of the basic biological mechanisms underlying DNA replication stress, and could prove clinically valuable for treating tumors where Rb and/or cyclin F are non-functional.

## Concluding Remarks

In summary, E2F1 is a critical player in the processes of DNA replication and cell cycle progression through its G_1_/S transcriptional activity and pro-apoptotic functions. E2F1 activity is normally restrained by feedback mechanisms, however when oncogenes are activated and/or tumor suppressors are inactivated E2F1-mediated transcription is unrestricted and DNA replication stress ensues (Lavoie et al., 1996; von Willebrand et al., 2003; Bartkova et al., 2005). In these settings, the DNA damage checkpoint kinases ATR and Chk1 are activated and can arrest the cell cycle to allow DNA repair or to promote the execution of apoptosis through E2F1 itself when the DNA insults are irreparable (Liu et al., 2000; Zhang et al., 2009; Bertoli et al., 2013a,b; Bertoli et al., 2016). When the DNA damage checkpoint fails or is inhibited, unrestrained E2F1 activity stimulates DNA replication progression in the absence of DNA repair. It was observed that after Chk1 inhibition, cyclin F prevents E2F1 hyper-activity by promoting its ubiquitin-mediated degradation (Burdova et al., 2019). The depletion of cyclin F after checkpoint inhibition restores E2F1 hyper-activity, which underlies a DNA replication catastrophe (Burdova et al., 2019). Laboratory evidence suggests the therapeutic exploitation of Chk1 inhibitors in cancers lacking Rb (Witkiewicz et al., 2018). It is possible to envision that the inhibition of cyclin F could be used to enhance the synthetic lethality between Chk1 inhibition and Rb loss.

In order to further mine the translational potential of these observations, more work is necessary to understand the mechanisms underlying DNA replication stress derived from E2F1 hyper-activity. Furthermore, specific biomarkers of E2F1 hyper-activity need to be identified. In addition, it will be critical to understand whether there is redundancy between Skp2 and cyclin F in regulating E2F1, or if Skp2 and cyclin F are controlling different pools of E2F1, for example to balance its pro-survival vs. pro-apoptotic functions. Finally, whilst Skp2 has been extensively targeted through chemical approaches (Lee and Lim, 2016), cyclin F inhibitors have not yet been developed.

## Author Contributions

SF worked on the main body of the review and collected the relevant literature. DH critically analyzed and reviewed the manuscript. VD'A organized the literature, provided insightful comments, and revised the work. All authors contributed to the article and approved the submitted version.

## Conflict of Interest

The authors declare that the research was conducted in the absence of any commercial or financial relationships that could be construed as a potential conflict of interest.

## Abbreviations

ADP, TDP, CDP, GDP, UDP: adenine, thymine, cytosine, guanine, uridine diphosphate
APC/C: anaphase promoting complex/cyclosome
ATM: ataxia-telangiectasia mutated kinase
ATR: ataxia telangiectasia and rad3-related kinase
BER: base excision repair pathway
Cdc: cell division control protein
Cdk: Cyclin-dependent kinase
Chk1/2: checkpoint 1/2 kinase
CMG: Cdc45-MCM-GINS
Cul1: Cullin 1
DBD: DNA-binding domain
Ddk: DBF4-dependent kinase
DDR: DNA damage response
DHODH: dihydroorotate dehydrogenase (quinone)
dNDP: deoxyribonucleotide diphosphate
dNTP: deoxyribonucleotide triphosphate
DP: dimerization partner protein
DSB: double-strand break
E2F1: E2F transcription factor 1
EGFR: epidermal growth factor receptor
ERK: extracellular signal-regulated kinase
Gab2: Grb2-associated binder 2
GDP: guanosine diphosphate
GEF: guanine nucleotide exchange factor
Grb2: growth factor receptor bound protein 2
GTP: guanosine triphosphate
HDAC1: histone deacetylase 1
HPV: human papillomavirus
ILK: integrin-linked kinase
IMPDH2: inosine monophosphate dehydrogenase 2
KAP1: Krüppel-associated box-associated corepressor 1
MAPK: mitogen-activated protein kinase
MCM: mini-chromosome maintenance
MTBP: MDM2 (mouse double minute 2)-binding protein
mTORC2: mechanistic target of rapamycin complex 2
NDP: ribonucleotide diphosphate
NME1: non-metastatic cells 1, protein (NM23A) expressed in
PDK-1: phosphoinositide-dependent protein kinase-1
PH: pleckstrin homology
PI3K: phosphoinositide 3-kinase
PIP_2_: phosphatidylinositol 4,5-bisphosphate
PIP_3_: phosphatidylinositol 3,4,5-triphosphate
PPAT: phosphoribosyl pyrophosphate amidotransferase
pre-RC: pre-replication complex
RASGEF1B: RasGEF domain family member 1B
RASGRP1: Ras guanyl releasing protein 1
Rb: retinoblastoma
RER: ribonucleotide excision repair pathway
RNR: ribonucleotide reductase
RRM1/2: ribonucleotide reductase member ½
RTK: receptor tyrosine kinase
SCF: Skp1–Cul1–F-box
SH2: Src homology 2
Sos: son of sevenless
ssDNA: single-stranded DNA
SWI/SNF: SWItch/sucrose non-fermentable
TopBP1: topoisomerase IIβ-binding protein
UPS: ubiquitin-proteasome system
UV: ultraviolet.
